# FACTORS INFLUENCING COGNITIVE FUNCTION AMONG THE US OLDEST-OLDER ADULTS

**DOI:** 10.1093/geroni/igad104.2624

**Published:** 2023-12-21

**Authors:** Jung Yoen Son, Janet Larson

**Affiliations:** University of Michigan, Ann Arbor, Michigan, United States; University of Michigan, Ann Arbor, Michigan, United States

## Abstract

Previous studies have identified factors that influence cognition in older adults (aged ≥ 65); however, the findings cannot be generalized to the oldest-old adults (aged ≥ 80), who face different physical and functional challenges. This study aimed to examine the association between potential influencing factors and cognitive function in community-dwelling oldest-older adults using the National Health Aging Trends Study (NHATS) dataset, a nationally representative sample from 2015. We conducted linear multilevel modeling (MLM) to examine the association between participation in physical activity (PA) and social activity, everyday technology use, mental health, and self-reported health with cognitive function (immediate and delayed word recall), adjusting for five sociodemographic characteristics (age, sex, race/ethnicity, and education). A total of 2779 community-dwelling oldest-old adults from the NHATS were included. Most of study participants were 80-84 years old (47%), female (61%), Non-Hispanic White (70%), not married (63%), and did not obtain a college degree (75%). In the MLM, participating in physical and social activities, using technology, and poorer mental health were significantly associated with higher word-recall scores (better cognitive function). Self-reported health was not associated with word recall. Results from our study suggest that interventions aimed at promoting physical and social activities and improving mental health may help maintain cognitive function among the oldest-old population. Our findings also suggest the potential positive impact of technology use on cognition, although further study is needed to support the evidence.

